# Uterine tumor resembling ovarian sex-cord tumor: case report and review of the literature

**DOI:** 10.2478/abm-2022-0018

**Published:** 2022-06-30

**Authors:** Rong Xu, Liping Shao, Wenling Zhang, Zhi-Long Yang

**Affiliations:** Department of Obstetrics and Gynecology, Nanjing Lishui People's Hospital, Nanjing, Jiangsu 211200, China; Department of Pathology, Nanjing Lishui People's Hospital, Nanjing, Jiangsu 211200, China; Department of General Surgery, Nanjing Lishui People's Hospital, Nanjing, Jiangsu 211200, China

**Keywords:** case reports, fertility preservation, immunohistochemistry, surgery, uterine neoplasms

## Abstract

**Background:**

We report the clinicopathological characteristics, immunohistochemical features, ultrastructure, tissue source, differential diagnosis, treatment, and prognosis of a patient with a uterine tumor resembling ovarian sex-cord tumor (UTROSCT).

**Case report:**

A 40-year-old woman had a uterine myoma with enlargement for 2.5 years. An ultrasound examination showed a mixed echogenic mass at the posterior wall of the uterus and a dark cyst in the right adnexal area, which suggested a suspected uterine myoma with liquefaction and a suspected chocolate cyst. The patient underwent transabdominal tumor resection with removal of the right adnexal mass. Through postoperative pathological examination, the patient was diagnosed with UTROSCT. No recurrence was observed after a follow-up of 1 year.

**Conclusion:**

Although UTROSCT is usually benign, it can relapse or metastasize, and patients with UTROSCT need comprehensive diagnosis and treatment.

Uterine tumor resembling ovarian sex-cord tumor (UTROSCT) is an extremely rare gynecological tumor with low malignant potential. To our knowledge, Clement and Scully [1] in 1976 were the first to report this uterine stromal neoplasm. Type II UTROSCT is solely composed of sex-cord components and is usually benign or shows low-grade malignancy with occasional local recurrence or distant metastasis [2]. However, recent studies have reported postoperative recurrence and lymphatic metastasis in some cases of UTROSCT [3, 4]. Some authorities proposed that UTROSCT should be categorized as a tumor with undetermined malignant potential. According to the World Health Organization (WHO) 2014 classification, UTROSCT was categorized as an endometrial stromal tumor [5]. Later, the WHO (2020) classification recategorized UTROSCT as a miscellaneous mesenchymal tumor and defined it as a uterine tumor with similar morphology to ovarian sex-cord tumors, lacking recognizable endometrial mesenchymal components [6]. Although there are several reports about UTROSCT in China and abroad [7, 8, 9, 10, 11, 12, 13, 14, 15, 16, 17, 18, 19], pathologists still lack sufficient understanding of its clinicopathological features. Here, we report a case of UTROSCT and retrospectively analyze its clinical and pathological characteristics. Additionally, a review of the literature reporting clinical cases of UTROSCT was conducted to understand better this rare gynecological tumor in clinical practice.

## Case report

A 40-year-old woman underwent a physical examination at our hospital in October 2019, and the gynecological ultrasound examination suggested a right adnexal mass (5.9 cm diameter) and uterine myoma (5.8 cm diameter) with partial liquefaction. She did not have abnormal symptoms, such as hypermenorrhea or a history of abnormal uterine bleeding, and denied a previous medical history of gynecological diseases, family history of malignancy, or history of hereditary syndromes or disorders.

**Figure 1:**
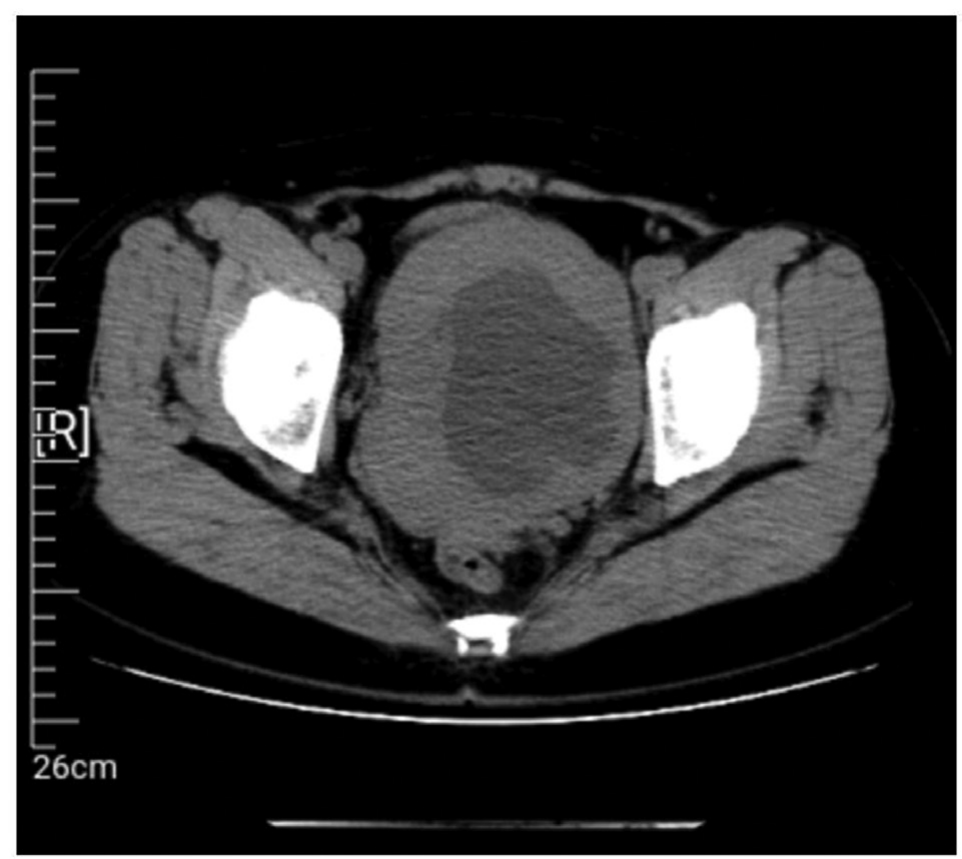
Pelvic computed tomography image showing an unclear defined 11 cm pelvic mass without further abnormalities.

**Figure 2:**
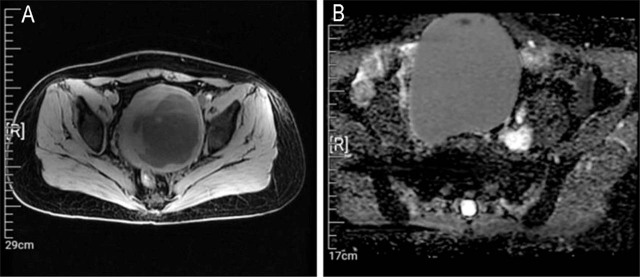
Magnetic resonance imaging and macroscopic findings. **(A)** T2-weighted image and **(B)** diffusion-weighted image depicting a high signal intensity in the uterine fibroid.

No regular ultrasound reexamination was performed until April 2021, and the results showed an enlarged and morphologically abnormal uterus, a mixed uneven echogenic mass at the posterior wall of the uterus (101 mm × 85 mm, with 7 mm thick endometrium), and a dark cyst (109 mm × 93 mm) in the right adnexal area with dense punctate echo, suggesting suspected uterine myoma with liquefaction and a suspected chocolate cyst. Abdominal computed tomography showed lesions in the pelvis and uterus (**Figure 1**). A pelvic magnetic resonance imaging examination suggested a suspected myoma cyst with malignant potential in the uterus and a suspected cystadenoma in the right adnexal area (**Figure 2**).

**Figure 3:**
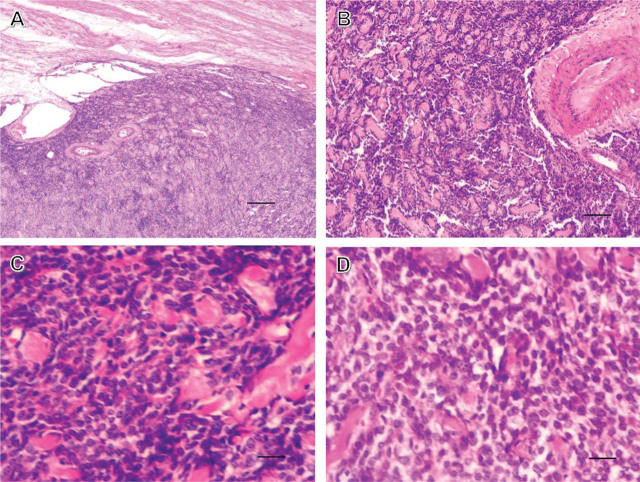
Dense long spindle cell configurations and hyaline stromal changes under hematoxylin and eosin staining. **(A)** 40× (scale bar 250 μm), **(B)** 100× (scale bar 100 μm), **(C)** 400× (scale bar 50 μm), and **(D)** 400× (scale bar 50 μm).

**Figure 4:**
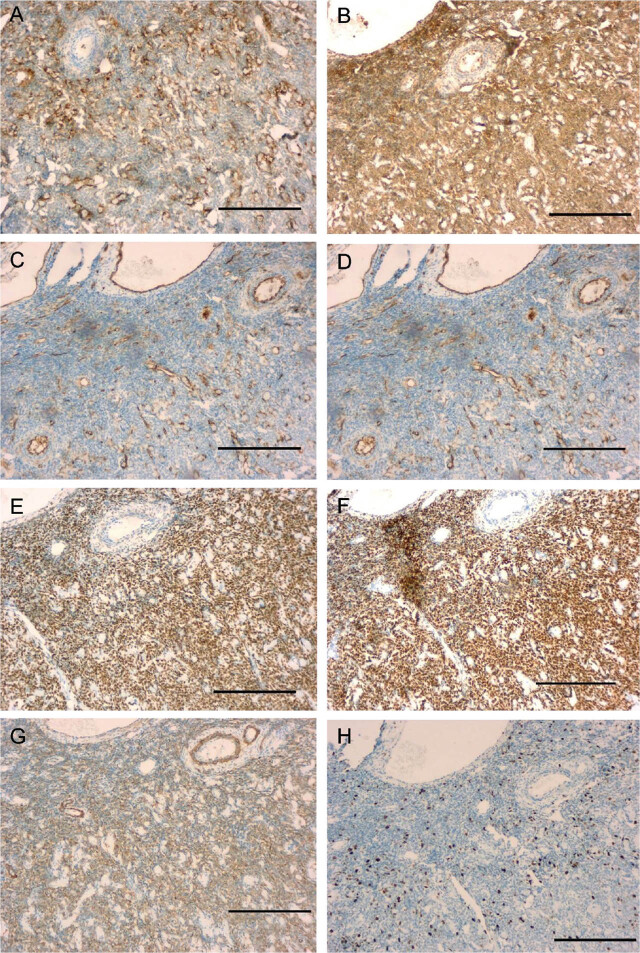
Immunohistochemistry of the UTROSCT. The tumor was positive for (A) CD10 (2+), (B) CD99 (3+), (C) CD34 (+), (D) desmin (3+), (E) ER (3+), (F) PR (3+), (G) SMA (3+), and (H) Ki67 (15%+); magnification: 100× (scale bar 100 μm). The antibodies used were as follows: anti-calretinin (catalog No. CCR-0221, Sainuote Biological Technology Co., China; 1:50); anti-CD10 (catalog No. C6D1; Sainuote Biological Technology; 1:50), anti-CD117 (catalog No. EP10, Sainuote Biological Technology; 1:50), anti-CD99 (clone: 013; catalog No. GT212302, Sainuote Biological Technology; 1:50), anti-CD34 (clone: QBend/10; Sainuote Biological Technology; 1:50), anti-D2-40 (catalog No. D2-40, Sainuote Biological Technology; 1:50), anti-desmin (catalog No. C3B7, Sainuote Biological Technology; 1:50), anti-ER (catalog No. FNab02822, Wuhan Feieng Biological Technology Co., China; 1:50), anti-PR (catalog No. YZK7169, Shanghai Yanzhun Biological Technology Co., China; 1:50), anti-SMA (catalog No. C1C1; Sainuote Biological Technology; 1:50), and anti-Ki67 (catalog No. C3G4, Sainuote Biological Technology; 1:50). ER, estrogen receptor; PR, progesterone receptor; SMA, α smooth muscle actin (actin α-2 (ACTA2)); UTROSCT, uterine tumor resembling ovarian sex-cord tumor.

Total hysterectomy and right adnexectomy were discussed based on the pathological conditions and examination results. However, the patient had a fertility requirement, and; therefore, was treated by transabdominal tumor total resection with the removal of the right adnexal mass 1 month later. The tumor was located at the left fundus of the uterus, with no clear boundaries. A little yellow liquid flowed during the resection of the tumor. No. 1 absorbable sutures were used for the interrupted suture of the uterus. The postoperative pathological examination suggested an endometrioid cyst in the right ovary, a uterine stroma–originated tumor with cystic degeneration, and negative margins of the resected specimens (**Figure 3**). Immunohistochemical results (**Figure 4**) showed calretinin (also known as calbindin 2) (−), CD10 (2+), CD117 (−), CD34 (blood vessel+), CD99 (3+), D2-40 (M2A antigen) (lymph vessel+), desmin (3+), estrogen receptor (ER) (3+), Ki67 (also known as MKI67) (15%+), progesterone receptor (PR) (3+) α smooth muscle actin (α-SMA) also known as actin α-2 (ACTA2) (3+), α-inhibin (−), and cytokeratin (−). A differential diagnosis suggested UTROSCT or uterine leiomyoma, which needed to be confirmed through immunohistochemistry and molecular pathology results. Pathological sections were sent to a higher level hospital, and the results showed a mesenchymal tumor in the uterus, the morphological and immunohistochemical features of which were consistent with those of UTROSCT. Immunohistochemical reports from that hospital showed caldesmon (−), CD10 (−) (anti-CD10; catalog No. C6D1; Sainuote Biological Technology Co., China; 1:50), cyclin D1 (−) (anti-cyclin D1; clone: EP12; catalog No. CCR-1142, Sainuote Biological Technology; 1:50), FOXL2 (+) (anti-FOXL2; catalog No. ab5096, Abcam; RRID: AB_304750; 1:50), and a-inhibin (−) (anti-inhibin; clone: EP378; catalog No. GT230202, Sainuote Biological Technology; 1:50). Additionally, a molecular pathological examination did not detect breakpoints in *JAZF1* (7p15) or *NCOA2*. The patient was discharged, and subsequent follow-up showed no abnormal symptoms or disease recurrence for at least 1 year after surgery. The present report was approved by the Scientific and Ethical Committee of Nanjing Lishui People's Hospital. Documented informed consent was obtained from the patient for publication of this case report and any accompanying images on a specified patient privacy and confidentiality form. This case report was prepared following the CARE Guidelines [20].

## Discussion

UTROSCT mostly affects reproductive and postmenopausal women with an average age of 48 years. The youngest case of UTROSCT was reported in a 22-year-old nonpregnant woman [7]. Common clinical manifestations of UTROSCT include abnormal vaginal bleeding and/or enlargement of the uterus. Some patients with UTROSCT may have pelvic pain or discomfort, while others lack typical clinical manifestations and do not seek medical attention until lesions are found in the uterus or pelvis accidentally through imaging examination. UTROSCT usually originates as a single polypoid myomatous nodular mass, and while it mostly grows in the intermural and submucosa of the uterus, reports of cervical UTROSCT are also found in the literature [3]. Our data described the clinical and pathological characteristics of a UTROSCT case.

By performing immunophenotyping of UTROSCT, a broad spectrum of expressed proteins was reported in previous studies, including coexpressed steroid hormone receptors, cytokeratin, smooth muscle markers, and positive markers in ovarian sex-cord–stromal tumors such as inhibin, calretinin, CD56, CD99, melan-A, steroidogenic factor 1 (SF-1), and FOXL2 [11]. The positively expressed calretinin ranks the highest in UTROSCT cases and is considered the most important marker for the differential diagnosis against other uterine lesions with sex-cord–like architectures (such as leiomyoma and leiomyosarcoma) [12, 13]. However, findings of negative expression of calretinin have also been reported in some cases of UTROSCT. Additionally, some studies suggest that the positive staining of SF-1 is an effective marker in distinguishing UTROSCT from other lesions, but the low expression rate of SF-1 might limit its usage. Among the 26 case series collected by Goebel et al. [14], the positive rate of inhibin was 69.6% (16/23), of which 5 cases had at least 1 positive-stained marker (any 1 or a combination among calretinin, WT-1, and CD56), and those with positive staining of inhibin were all AE1/AE3-positive (8/8) and desmin-positive (12/12). The positive expression rate of hormone receptor SF-1 was 81.8% (9/11) and that of PR was 100% (9/9). In the present case report, immunohistochemical findings excluded endometrial stromal tumor and smooth muscle tumor components. Meanwhile, based on the hematoxylin and eosin staining of tumor sections of the patient and the diagnosis criteria of 2020, the patient was ultimately diagnosed with UTROSCT.

The molecular pathogenesis of UTROSCT remains unclear. Studies have shown that *NCOA2/3* fusion is a characteristic of UTROSCT [11, 15], serving as a diagnostic indicator. Dickson et al. [15] performed transcriptome sequencing in 4 patients who were pathologically diagnosed with UTROSCT and found that 2 patients had the *ESR1*-*NCOA3* fusion gene and the other 2 had *ESR1*-*NCOA2* and *GREB1*-*NCOA2* fusion genes. Some studies have also detected other gene fusions in cases of UTROSCT, such as the *GREB1*-*NCOA1* and *GREB1-CTNNB1* fusion genes [14]. UTROSCT is a unique neoplasm characterized by recurrent *NCOA1*-*3* fusion genes [14]. *NCOA* encodes a series of nuclear hormone receptor coactivators like *NCOA1*-*3*, which stimulates transcription in a hormone-dependent manner after binding to nuclear receptors. Their hormone-dependent uterine function and dysfunction have been validated in a mouse model of p160/steroid receptor coactivator (SRC) expression and endometrial biology and pathobiology [21]. Several solid tumors are histologically similar to UTROSCT, including endometrial stromal tumors with sex-cord components, endometrioid carcinomas with sex-cord growth, epithelioid smooth muscle tumors, and perivascular epithelioid cell tumors. In particular, specimens collected from endometrial biopsy, curettage, or myomectomy enhance the difficulty in the differential diagnosis of UTROSCT. Among methods for the differential diagnosis of UTROSCT against other tumors, molecular detection seems the most promising; however, considerable time may elapse before a complete functional understanding of the molecular profiles of these tumors can be obtained and molecular tests are relatively expensive [12].

While total hysterectomy with bilateral adnexectomy is the most common treatment for UTROSCT, the treatment needed should be considered specifically on a case-by-case basis, because age and fertility requirements play an important role in determining whether these surgeries are appropriate. Zhang et al. [3] had reported 2 cases of UTROSCT, and no recurrence was observed in the 12-year follow-up of the 33-year-old patient after total hysterectomy without reproductive requirement and the 1-year follow-up of the 64-year-old patient after total hysterectomy with bilateral adnexectomy. Jeong et al. [16] reported a 32-year-old patient with UTROSCT and intramuscular infiltration who conceived after hysteroscopic resection of the tumor. Sato et al. [4] reported a 57-year-old patient with UTROSCT and sarcoma features who initially underwent transabdominal total hysterectomy with bilateral adnexectomy, and then pelvic and abdominal paraaortic lymphadenectomy and subtotal omentectomy, based on postoperative pathology. The patient did not show recurrence within 36 months after surgery. They concluded that old age, necrosis, lymphatic vascular invasion, significant nuclear atypia, and significant mitotic activity are high-risk factors for malignant UTROSCT, and extensive radical surgery is required to prevent the recurrence and metastasis of UTROSCT. Moore and McCluggage [17] reviewed the follow-up data and prognosis of UTROSCT with the largest sample size and proposed that UTROSCT with malignant progression usually has necrosis and a mitotic rate ≥1 per 50 high-power fields. Carbone et al. [18] reported 2 patients with UTROSCT who underwent conservative surgery, with subsequent successful pregnancy outcomes, although the study suggested that radical resection should be considered after childbirth. Watrowski et al. [10] reported a patient with UTROSCT who received hysteroscopic surgery with a recurrence-free survival of at least 28 months. De Franciscis et al. [19] reported a patient with UTROSCT who received resectoscopic surgery with successful delivery and a recurrence-free survival of at least 60 months. Minimally invasive surgery might be a feasible option for patients with UTROSCT and fertility requirements; however, this view still needs to be empirically substantiated with more data with long-term follow-up.

## Conclusion

UTROSCT is a very rare gynecological tumor. The diagnosis and differential diagnosis of UTROSCT rely mainly on pathological examinations. Therefore, it is necessary to investigate further the histological characteristics, immunophenotypes, and long-term biological behaviors of UTROSCT to improve our understanding of this rare tumor.
